# Personalized brain stimulation for effective neurointervention across participants

**DOI:** 10.1371/journal.pcbi.1008886

**Published:** 2021-09-09

**Authors:** Nienke E. R. van Bueren, Thomas L. Reed, Vu Nguyen, James G. Sheffield, Sanne H. G. van der Ven, Michael A. Osborne, Evelyn H. Kroesbergen, Roi Cohen Kadosh

**Affiliations:** 1 Wellcome Centre for Integrative Neuroimaging, Department of Experimental Psychology, University of Oxford, Oxford, United Kingdom; 2 Behavioural Science Institute, Radboud University Nijmegen, Nijmegen, the Netherlands; 3 Department of Materials, University of Oxford, Oxford, United Kingdom; 4 Department of Engineering Science, University of Oxford, Oxford, United Kingdom; 5 School of Psychology, Faculty of Health and Medical Sciences, University of Surrey, Guildford, United Kingdom; 6 Amazon, Adelaide, Australia; University of Nottingham, UNITED KINGDOM

## Abstract

Accumulating evidence from human-based research has highlighted that the prevalent one-size-fits-all approach for neural and behavioral interventions is inefficient. This approach can benefit one individual, but be ineffective or even detrimental for another. Studying the efficacy of the large range of different parameters for different individuals is costly, time-consuming and requires a large sample size that makes such research impractical and hinders effective interventions. Here an active machine learning technique is presented across participants—personalized Bayesian optimization (pBO)—that searches available parameter combinations to optimize an intervention as a function of an individual’s ability. This novel technique was utilized to identify transcranial alternating current stimulation (tACS) frequency and current strength combinations most likely to improve arithmetic performance, based on a subject’s baseline arithmetic abilities. The pBO was performed across all subjects tested, building a model of subject performance, capable of recommending parameters for future subjects based on their baseline arithmetic ability. pBO successfully searches, learns, and recommends parameters for an effective neurointervention as supported by behavioral, simulation, and neural data. The application of pBO in human-based research opens up new avenues for personalized and more effective interventions, as well as discoveries of protocols for treatment and translation to other clinical and non-clinical domains.

## Author summary

The common one-size-fits-all approach used in biological and behavioral research has shown to be inefficient. This is especially the case in the field of brain stimulation, where many different combinations of stimulation parameters (i.e., frequency and current strength of the applied current) can be used for restorative or enhancement purposes, in clinical and non-clinical populations, respectively. Even intervention protocols that have reported to be effective for certain individuals can be detrimental for others. Here we present an active machine learning method, personalized Bayesian optimization (pBO) that successfully searches, learns, and recommends neurostimulation parameters across individuals. Based on an individual’s baseline cognitive ability, the pBO identifies specific combinations of transcranial alternating current stimulation parameters, which are most likely to improve cognitive performance, in which case arithmetic problem solving. This timely approach provides a possible solution for the pressing need for personalization in different disciplines including medicine, psychology, and education.

## Introduction

There is no doubt that the human organism is complex, and the impact of nature and nurture, as well as their interaction, increases variability between humans. It is therefore not surprising that interventions aimed at altering human behavior are not effective for all individuals. This variability in effectiveness is partly due to the one-size-fits-all approach that currently dominates behavioral intervention research. Accumulating evidence has indicated that this approach is inefficient, and that a treatment that benefits one individual can be ineffective or even detrimental for another individual [1–8]. Personalized medicine aims to address this challenge by adjusting treatments to the individual or to a subset of patients [9,10]. Due to the complexity of individual differences, there is an increasing need for personalized medicine for a wide range of drugs, biomedical treatments, and diseases. Without this, the one-size-fits-all approach often only alleviates symptoms in clinical studies without curing the disease [11]. This demand for personalization is especially true in the field of transcranial stimulation, where electrical currents targeting specific brain regions are used to alter behavior. Whilst tailoring a stimulation protocol is ideal, identifying the optimal stimulation protocol for an individual proves problematic in large parameter spaces, where the systematic testing of each parameter combination can lead to overly costly and time-consuming protocols. For instance, one stimulation technique that is gaining popularity is transcranial alternating current stimulation (tACS) [12]. tACS utilizes an alternating current delivered via multiple electrodes placed on the scalp, which is capable of propagating through the scalp and modulating the activity of the underlying neurons. The applied alternating current promotes oscillatory activity at the stimulation frequency [13], allowing direct modulation of brain oscillations that subserve cognitive processes [14]. Through this process, tACS provides an attractive way to investigate causal predictors of behavior and to use such knowledge to improve human capabilities or health. However, exploring the effects of all tACS parameters on the performance of different individuals requires an exhausting amount of testing when considering different current (0–2 mA) and frequency (0–100 Hz) combinations.

One recently proposed method for selecting parameters in brain stimulation is Bayesian optimization (BO) [15,16]. BO is an active machine learning technique that aims to find the global optimum of a black-box function *f*(*x*) by making a series of evaluations. To select the next evaluation, BO first constructs a probabilistic model (surrogate model) for *f*(*x*) and exploits this model to make decisions. This results in a procedure that can find the maximal value of difficult non-concave functions with relatively few evaluations, at the cost of performing more computations to determine the next point (at minimal cost when compared with the effort of evaluating the function at more points) [17]. Hence, BO is particularly valuable when there is a need to explore a large experimental space in as few evaluations as possible. Generally, BO involves two procedures: 1) fitting an appropriate model to function *f*(*x*) and 2) choosing an acquisition function α(x) that steers sampling in the direction where improvement over the current best evaluation is most likely. It should be mentioned that other brain state-dependent stimulation methods that included personalized real-time electrophysiological (EEG) correlates show promising results [18].

**Fig 1:**
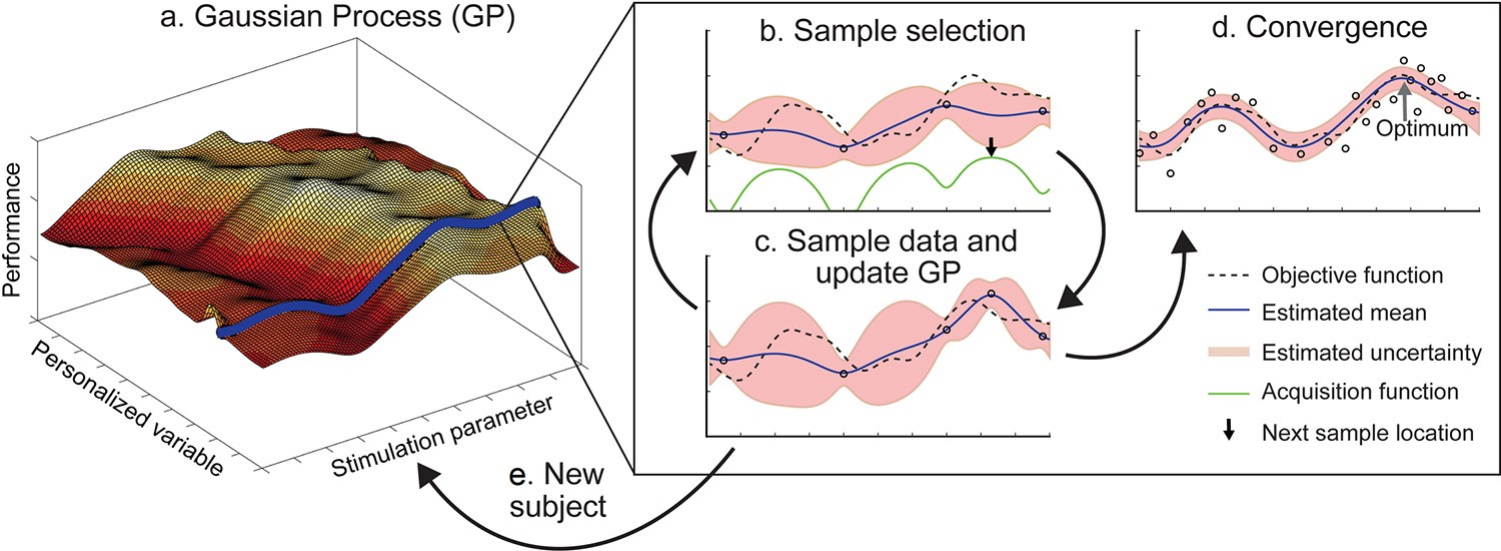
Illustration of the personalized Bayesian optimization procedure of theoretical values. **a)** The Gaussian process (GP) is fitted to the existing data and models the expected performance along parameter and personalized dimensions. **b)** The acquisition function identifies the next point to evaluate along the value of the personalized variable relevant to the participant. **c)** Once the data is collected at this new point the GP is updated and a new point selected. **d)** This cycle continues until either a new subject is tested, in which case a different value for the personalized variable will be recorded. **e)** a pre-set stopping criterion is reached, such as the number of subjects to be tested; or until the potential improvement is considered negligible (convergence). In this study, we utilized a pre-set stopping criterion of 50 subjects, after which testing was ceased.

The present work was inspired by previous work that used BO in human-based research [15,16,19–21]. In these previous BO studies, all iterations of the process are run on the same individual, allowing the experimenters to achieve person-specific results. However, to do this, the entire BO process must be run for each individual that requires stimulation—a lengthy and costly process. A workable solution is to base the algorithm on a measurable characteristic that varies across subjects, such as baseline ability in the behavioral task of interest. Therefore, we developed a novel personalized (p)BO for human-based research. In pBO, the algorithm is trained on an initial small set of data (burn-in phase), and then iteratively selects stimulation parameters across subsequent subjects, with the aim of identifying the optimal stimulation parameters for improving behavioral performance, whilst considering personalized information. i.e. baseline arithmetic ability (**Fig 1**).

This BO algorithm can incorporate personalized information [22], including an individual’s data, such as age, gender, neural activity or cognitive profile. Based on the vast literature that highlights the impact of individual differences on stimulation efficacy [23,24], we personalized the BO to subject’s cognitive ability in this study. We focused on optimizing arithmetic performance considering its importance in the success of one’s future career and socio-economic status [25] and its impairment in acquired and congenital brain disorders [26]. Skills needed for solving arithmetic problems vary greatly in the typical and atypical populations [27,28]. Similarly, a recent study on arithmetic skills highlighted the individual differences in both neural correlates and behavioral response in healthy people [29]. The left frontoparietal network has been implicated in playing an important role in arithmetic processing and can be targeted by tACS [30,31]. We recognize that other brain stimulation techniques have been used in the field of arithmetic [32–37], for reviews see [31,38,39]. However, we utilized tACS since this method allows for stimulation at a range of specific frequencies to explore those that might impact arithmetic performance.

**Fig 2:**
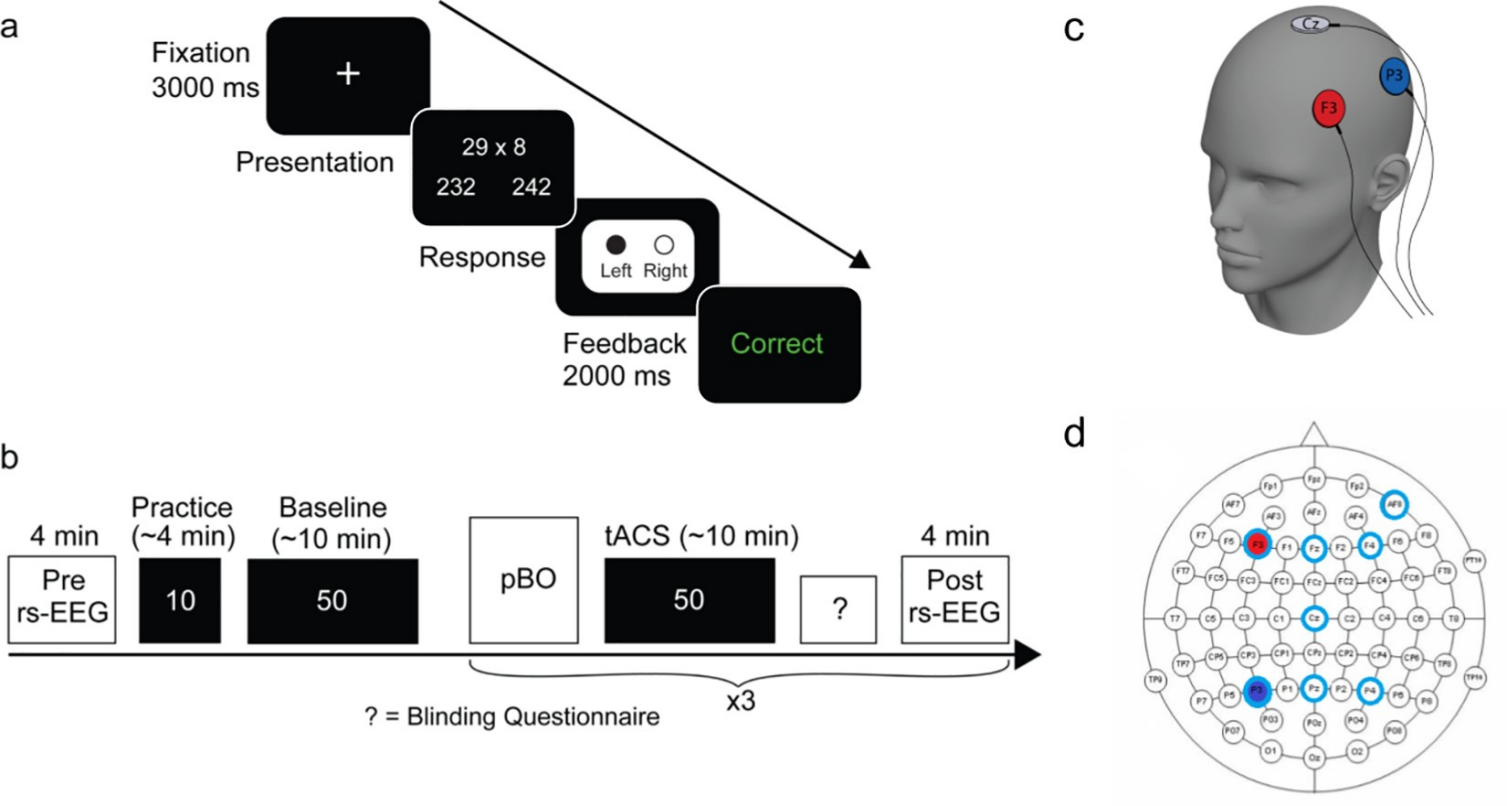
An overview of the experimental paradigm. **a)** An overview of the behavioral paradigm. Subjects (n = 50) watched a fixation point that indicated the start of a trial. After 3000 ms an arithmetic multiplication was shown with two possible answer options on the left and right side with a difference of 10 to keep consistency in task difficulty. Subjects responded by pressing either the left or right button on a response box as quickly and accurately with no time limit present. Lastly, subjects received either ‘correct’ or ‘incorrect’ as feedback to continuously capture attention. **b)** Subjects first completed a baseline rs-EEG of four minutes, after which 10 practice trials of multi-digit times single-digit multiplications were presented of four minutes. This was followed by the baseline task of 10 minutes, which comprised five blocks of 10 different multiplications. Subjects had a short break (~3 minutes) between baseline and the pBO. Three different tACS frequency-current combinations were proposed by the pBO algorithm after the completion of each sequence of 50 trials of the multiplication task which was approximately 30 minutes in total. Between these tACS combinations, post-block rs-EEGs of four minutes were recorded before the subjects moved on to the next tACS combination. Validation of the blinding of the stimulation and perceived sensations were assessed after completion of a stimulation block. **c)** An illustration of the tACS electrode montage. Stimulation was applied over the left frontoparietal area (F3 and P3) with one return electrode (Cz). **d)** A top down topoplot showing both the stimulation electrodes (red and blue) and the EEG electrode placing (turquoise).

We examined whether we could tailor tACS parameters to improve arithmetic performance using a pBO that takes baseline ability into account in healthy subjects. To do this, the individual’s baseline arithmetic ability was initially measured, after which the stimulation parameters to be used were automatically selected either at random, if subjects were in the initial burn-in phase (initialization phase), or by the pBO algorithm. Subjects then completed a block of the arithmetic behavioral task whilst receiving stimulation using the selected parameters (**Fig 2**). Stimulation parameter selection and behavioral testing were repeated in each subject until three blocks of different arithmetic problems were completed. Note that these three blocks were included to select more samples to allow optimization based on the pBO across subjects, rather than optimizing performance over these three iterations. The tACS parameters that were altered were current intensity and frequency, and the pBO algorithm aimed to identify the optimal parameter combination for improving arithmetic ability given a subject’s baseline arithmetic ability. To target the ability to solve arithmetic problems more precisely we used diffusion modeling, which allowed us to incorporate human performance while taking into account measures of both accuracy and reaction time in its calculation, a measure of cognitive ability, rather than auxiliary components such as non-decision response time or response conservativeness [40]. Furthermore, we ran different computational simulations to demonstrate the efficiency of our proposed pBO in comparison to random sampling and a standard BO algorithm (i.e., pBO without a personalized variable). We also recorded electrophysiological frequency band power and connectivity at baseline and after applying combinations of tACS parameters to link behavioral changes to EEG outcomes, while we report this finding, we note that it is not the main focus of the present study.

## Results

### Personalized Bayesian optimization

**Fig 3:**
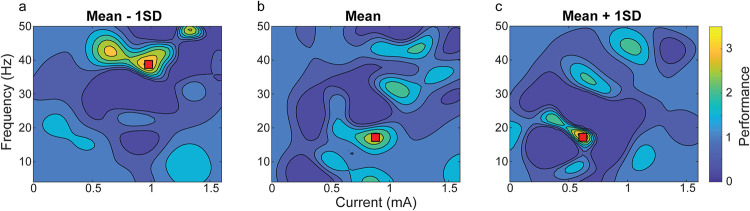
Results of the personalized Bayesian optimization model at several different baseline abilities (n = 49). The figure shows the predictions from the Gaussian process model for low baseline ability (panel a: 1 standard deviation (SD) below the mean), mean baseline ability (panel b: mean = 0.055), and for high baseline ability (panel c: 1 SD above the mean). The y-axis shows the frequency range of the applied stimulation (0–50 Hz) and the x-axis the current of the stimulation (0–1.6 mA, peak-to-peak). Arithmetic performance is indicated in color based on the normalized drift rates (tACS block/baseline block). Low drift rates are shown in dark blue and high drift rates in yellow. A best-inferred point for arithmetic performance according to a specific frequency-current combination is indicated by a red square in all three panels. Note that this figure is not based on different groups of participants as in moderation analysis, but represents a three dimensional view of the GP’s surrogate surface at three different points for visualization purposes.

Baseline ability is a continuous parameter and it should be considered that the best inferred tACS combination differs along this continuum. Following the suggestion of Aiken, West, and Reno [41] to allow for further visual inspection, we visualized the continuum at three different points: low (mean -1 SD), mean and high (mean +1 SD) baseline ability. As **Fig 3** shows, the optimal stimulation parameters depended on the participants’ baseline ability: we found a shift from higher frequencies and currents in lower (poor) baseline abilities, to lower frequencies and currents in higher (better) baseline abilities (mean - 1SD (38.67 Hz, 0.97 mA), mean (16.67 Hz, 0.88 mA), mean + 1SD (18 Hz, 0.6 mA), mA values are peak-to-peak).

**Fig 4:**
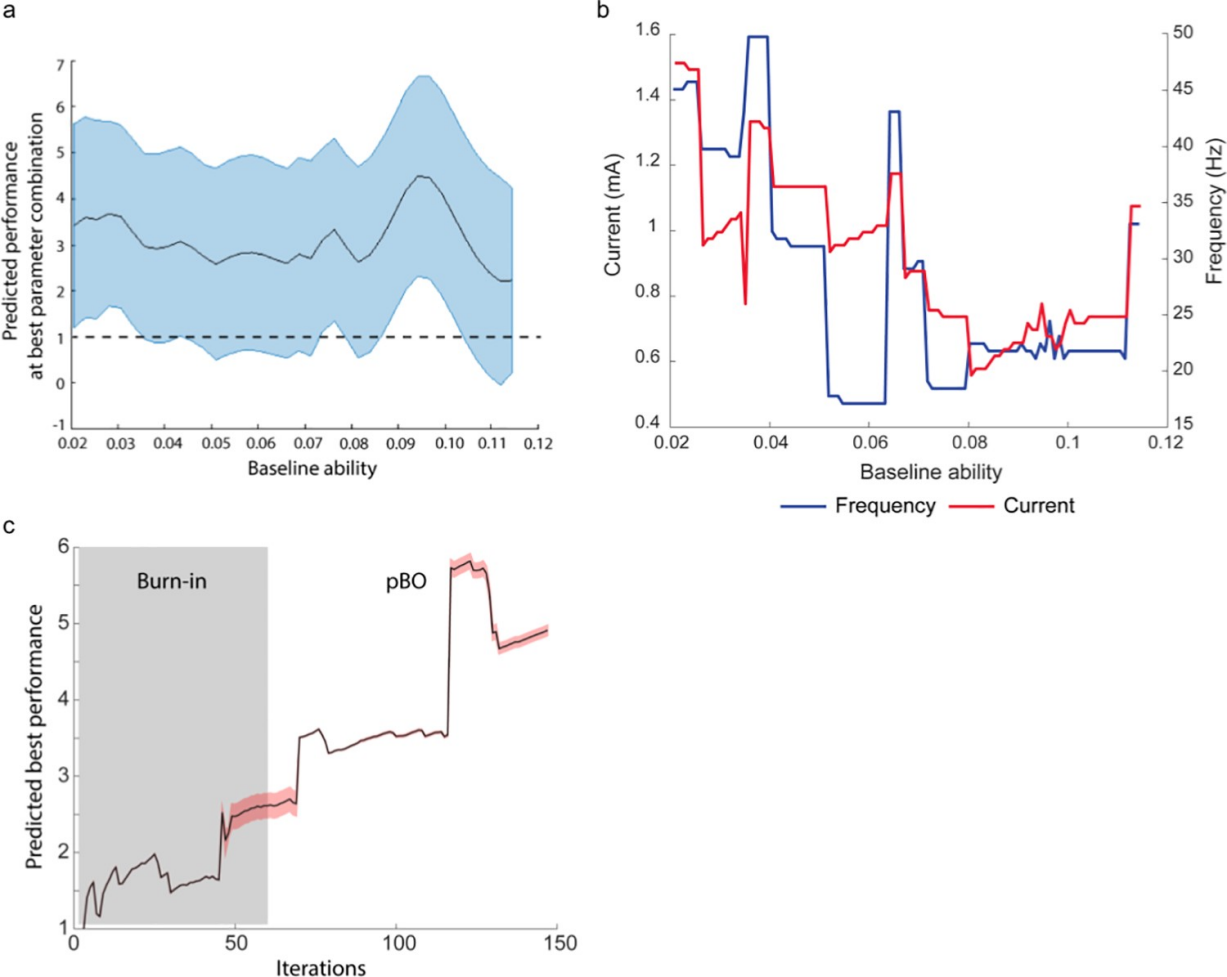
Results of optimizing behavior with personalized Bayesian optimization (pBO) (n = 49). **a)** In-depth visualization of the normalized performance according to baseline ability during pBO. Normalized performance was calculated as the drift rate of the performance block divided by the drift rate of the baseline block. Subjects on the lower part of the baseline ability spectrum showed a similar arithmetic performance improvement during tACS compared to subjects on the higher baseline ability spectrum. Note that a normalized performance score of 1 indicates no difference with baseline arithmetic performance when no stimulation was applied. A normalized performance score higher than 1 indicates improved performance as measured with drift rate. The blue shaded area indicates 95% credibility intervals. **b)** The change in frequency-amplitude tACS parameters proposed by the pBO algorithm based on the individualized baseline ability in arithmetic at the end of optimization. **c)** Predicted best performance at each iteration (i.e., different blocks), calculated as the best performance predicted by the GP at any parameter combination. Subjects were added sequentially, with three subsequent iterations were assessed for each participant. For example, iterations 148–150, represents blocks 1–3 for the 50^th^ subject. Surrogate uncertainty is shown by the shaded area in pink. Note that during some iterations uncertainty is higher due to new baseline abilities introduced in the pBO and due to outliers. These outliers are retested later which then reduces uncertainty.

A more in-depth visualization of the efficacy of the pBO procedure revealed that the overall fluctuation in performance improvement (e.g., normalized to the baseline performance without stimulation) across subjects with low and high baseline abilities was similar (**Fig 4A**). This result indicates that the success of our approach is equally effective for people with either low or high arithmetic baseline ability. The optimal frequency-current tACS parameter combinations proposed by the pBO algorithm confirms a shift from higher frequencies and currents in low-baseline ability subjects to lower frequencies and currents when baseline ability increases (**Fig 4B**; see also **Fig 3**). The behavior of the pBO algorithm can be seen in **Fig 4C** as the predicted best performance dramatically increases a number of times over the course of one iteration. This is where a beneficial stimulation combination is identified. Further, the predicted best performance tends to decrease soon after as that point is retested, and a more accurate estimation of the performance is identified. The best performance predicted by the GP was calculated as the highest value (i.e. “the best”) that the GP predicted across the frequency-current combinations. In addition, the black-box function *f*(x) is reliably optimized over the course of the iterations, as shown by an increase in the individual’s ability to solve arithmetic problems (**Fig 4C**).

Based on the suggestion of one of the anonymous reviewers, we also performed cross-validation as a resampling procedure to evaluate our pBO algorithm on a limited data sample. We performed cross-validation by randomly splitting 80% of the participants into a training group and the remaining 20% into a testing group. In this regression problem, the input included the current intensity, frequency and baseline ability. The output was the performance score in drift rate. The aim was to estimate how the model was expected to perform in general when used to make predictions on data not used during the training of the model. Using the Gaussian process for regression, we measured the Mean Squared Error (MSE) which is the average error between the true value against the predicted value. The results of this analysis showed an MSE of 0.3 for this validation task. To put this MSE score into context, the (arithmetic) performance score from this experiment ranges from 0.5 to 3.8 with a standard deviation of 0.54. We can see that the MSE of 0.3 is reasonable given the unavoidable measurement noise which is common in the field of experimental psychology.

### Personalized Bayesian optimization simulation analysis

**Fig 5:**
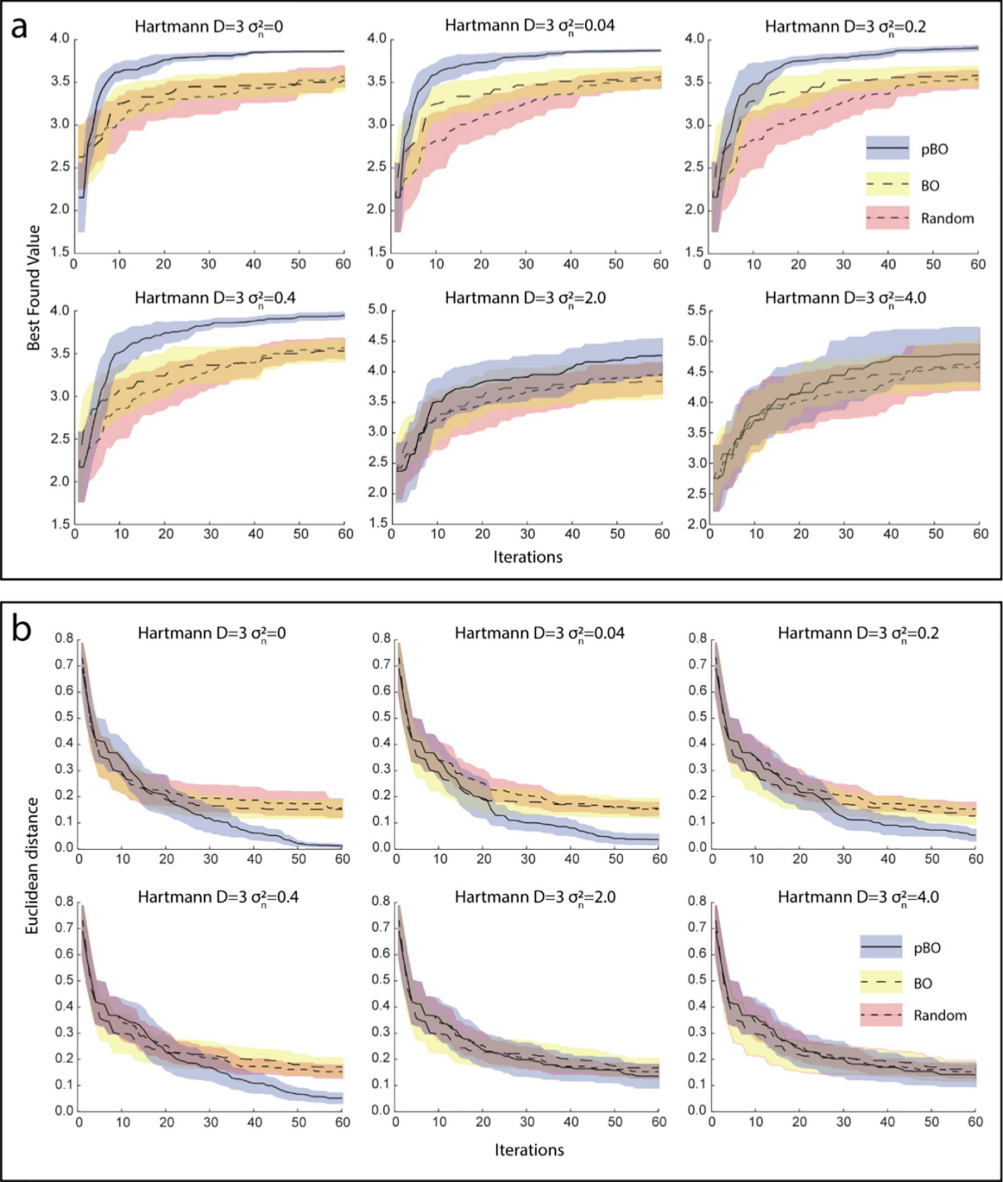
Results of simulating the ability of pBO, BO and random search algorithms on identifying the optima in the Hartmann 3-dimensional surface. The simulation was run 30 times on each of the six different levels of noise, lines represent the mean performance and shaded areas the standard deviation of 30 repeats. **a)** Shows the best found value identified by each algorithm at each iteration, demonstrating that the pBO algorithm is able to find higher values more quickly than the BO and random search algorithms. **b)** Shows the Euclidean distance of the identified optima from the true optima of the Hartmann function (i.e., accuracy of the algorithm). The pBO algorithm is shown to be more accurate than the BO and random search algorithms, except at very high levels of noise, where they are comparable.

To demonstrate the efficiency of our proposed pBO, we examined the optimization performance on a Hartmann 3-dimensional function [42]. This 3-dimensional function is a suitable benchmark representing our real experiments including three variables (frequency, current, and baseline ability). When running a Hartmann 3-dimensional optimization using the expected improvement (EI) [43] as an acquisition function in the pBO algorithm, the pBO algorithm outperformed a standard BO algorithm as well as random sampling (**Fig 5**). Whilst this is a comparison of a model with 3 parameters (pBO) outperforming a model with 2 parameters (BO), this simulates the additional data incorporated into the pBO model in the form of a relevant personalized variable, which is not present in standard BO. The results of this comparison show that higher drift rate values are attained more quickly when using the EI pBO procedure in comparison with BO and random sampling (**Fig 5A**), and the pBO algorithm was shown to identify an optima closer to the true optima of the Hartmann function (**Fig 5B**). When the noise variance $\sigma_{n}^{2}$ increases, the pBO performance is closer to the performance of random sampling and standard BO (**Fig 5**). As further mentioned in section ‘Acquisition function’ relating to hyperparameter considerations, the estimate of $\sigma_{n}^{2}$ from our observed data which varies by iterations ranged between 0.01 and 2.

Thus, if the behavioral evaluations of the experimental procedure are too noisy, the pBO procedure’s ability to make correct judgements about the optimum parameters is diminished but it is still able to outperform random sampling. Critically, as **Fig 5** illustrates within these estimated noise variance ranges, our pBO leads to improved optimization compared with the standard BO approach that does not take baseline ability into account. In particular, the standard BO is unable to enhance performance, thus highlighting the benefit of personalization vs. the one-size-fits-all approach.

### Baseline electroencephalography and arithmetic ability

To examine whether our results are supported by neurophysiology we used baseline electroencephalography (EEG). Previous EEG studies suggest a positive relationship between left frontoparietal theta (4–8 Hz) connectivity and high-level cognitive processing [44–48]. However, none was found when running a regression model trying to predict baseline arithmetic ability from baseline left frontoparietal connectivity in the theta range (all *p* > .3). The same applied to frontal theta power (*p* = .88), theta/beta ratio, and beta (14–30 Hz) connectivity (all *p* > .1). However, our findings from the pBO models highlight an optimal performance effect in the beta frequency range (14–30 Hz) in subjects with average and high baseline ability, whilst low baseline ability individuals benefit from stimulation in the gamma frequency range (> 30 Hz). We therefore examined the relationship between baseline ability and baseline frontal beta power in an exploratory manner. Higher (gamma) frequencies could not be recorded reliably with the equipment. We found that subjects with higher arithmetic baseline ability have higher baseline beta power in comparison to subjects with lower arithmetic baseline skills (non-parametric (Spearman) correlation: *r*_*s*_ = .29, *p* = .03). This neurophysiological finding corroborates the group-level pBO model results, where the pBO algorithm chose the tACS frequency that mirrors baseline neurophysiological activity in those individuals.

## Discussion

Most interventions in humans are not tailored to the individual’s characteristics, such as behavior or brain function, but use a one-size-fits-all approach that leads to inefficient or even ineffective interventions [1–8]. This lack of progress is rooted mainly in the complexity of personalizing interventions, due to the immense burden on time and resources. The results of the empirical and simulation experiments performed in the present study demonstrate that our pBO algorithm is capable of tailoring different current strengths and frequencies of tACS to an individual’s baseline ability. Specifically, we demonstrated that the optimal stimulation parameters, determined by the pBO algorithm, differ in low and high arithmetic baseline individuals. This suggests that there are either different cognitive processes involved or differing effects of stimulation in these groups. For example, a recent multiplication study indicated possible behavioral constraints of a one-size-fits-all brain stimulation protocol on performers in a certain subgroup of the population (e.g., high performers) [48]. Notably, the present results cannot be explained by a placebo (sham) stimulation, as we controlled for placebo effects of stimulation by including 0 mA in the search space of the pBO algorithm, as well as other very weak currents that are assumed to be ineffective. In addition, if our effects could be explained by a placebo effect, it should have led to the parameters that yielded the strongest sensation, which was not the case (**S2** and **S4 Figs**). Similarly, our results cannot be attributed to the participants learning effects as improvement in arithmetic performance was based on an improvement across participants, and not within.

Highlighting the significance of our results, the majority of previous brain stimulation studies that aimed to determine stimulation parameters have only tested a small number of different parameters to observe their differential effects [31]. However, this approach leaves a large amount of stimulation parameter combinations unexplored, as such exploration is both expensive and time consuming. Whilst a small number of studies have utilized BO, they have focused on running the entire BO paradigm on one individual to find their best stimulation parameters [15,16]. This approach, while providing many advantages, does not allow for the convenient transfer of parameters optimized for one subject to new subjects, and does not allow its usage in contexts that do not permit repeated sampling in the same individual due to ethical constraints, potential side effects, or time pressure. Additionally, the need to sample to the parameter space during the initial burn-in phase increases the sample size required to identify optimal stimulation parameters. In contrast, our pBO algorithm provides further advancements by including the following novel processes. Firstly, our algorithm receives personalized data, in this study baseline cognitive data from a subject, and suggests the stimulation parameters to test that are conditioned on the baseline data. Due to the ability to recommend personalized stimulation parameters solely based on a baseline measure, our work on a pBO algorithm represents a significant advance in this area. To illustrate, our experimental findings demonstrate that pBO preferentially selects more successful tACS parameters to optimize the interventional outcome, in this case arithmetic performance (**Fig 4**). Secondly, our study shows that non-personalized interventions, as in standard BO, are ineffective due to the inability to optimize performance effectively (**Fig 5**). Our simulations further show that pBO is reliable even when there is a considerable amount of noise present in the models. Previously, most BO applications have been in a noise-free context, in contrast with human-based studies that are prone to noisy evaluations. More precisely, as noise increases, the pBO algorithm is less able to evaluate the stimulation parameters correctly, but still outperforms random sampling and the standard BO algorithm. This furthermore applies to the estimated noise variance ranges that were observed from our data.

In addition, the behavioral and simulation results support our electrophysiological evidence that pBO can provide new protocols for intervention as well as mechanistic insights. In the present study, pBO highlighted the importance of frontal beta frequencies (14–30 Hz) as indicated by the BO group-level model in subjects with average and high arithmetic abilities. In line with our group-level pBO models, we showed that high baseline ability subjects have higher frontal beta power in comparison with low baseline ability subjects. In addition, subjects with low baseline abilities benefit more from tACS in the gamma frequency range, which is in line with responses linked to spike timing dependent plasticity (STDP) [49]. However, gamma frequencies could not be recorded reliably with EEG due to low-pass filtering properties of the skin and skull together with a low signal-to-noise ratio [50], and was therefore not statistically explored. This limitation can be overcome by magnetoencephalography (MEG), as was shown by a study indicating frequency-specific neural entrainment by tACS [51]. The overlap of our pBO model with the baseline EEG correlates provides a causal inference of the involvement of baseline oscillatory brain activity (notably beta activity) in mathematics performance, a relation that was only known to be correlational in the field of mathematical cognition until now [31,52].

One constraint of this approach that should be considered, is that the distribution of subject baseline abilities in this study was weighted towards the lower end of the ability range, leading to fewer subjects with higher baseline ability being tested (**S1 Fig**). Therefore, results in the lower baseline ability spectrum are of higher confidence regarding an estimation of the optimum frequency-current tACS combination. This notion is especially relevant on account of the similar results in our group-level pBO model for the mean and high baseline abilities. Additionally, the subjects that participated were mainly university (under)graduates, which might have led to a small arithmetic ability range when compared to the population. A possibility exists that our pBO models will differ slightly when testing more subjects with higher baseline abilities. Moreover, while we demonstrated the personalization of intervention based on two dimensions (i.e., the current and frequency of tACS) and a personalized feature, our algorithm allows for the inclusion of many more dimensions (e.g., phase, brain region, and duration of stimulation) in future interventions. Additionally, the personalized variables are pervasive in human-based research and can include multiple variables such as behavioral data, neural activity, age, and gender. For example, the presented findings could translate to other neurointerventions, such as other forms of transcranial electrical stimulations, transcranial magnetic stimulation (TMS) or to sensing-enabled brain stimulation such as deep brain stimulation (DBS) and the responsive neurostimulation system (RNS). Similar to tACS, these interventions use a broad range of stimulation parameters whereby it is uncertain which parameters are more successful to optimize the interventional outcome due to individual differences in healthy subjects or patients [53–55]. Our pBO approach overcomes this limitation by personalizing the intervention based on the selection of stimulation dimensions together with a personalized feature. Taken together, the use of our pBO approach is widely applicable, and can simultaneously model multiple dimensions together with a wide range of choices of personalized variables. Further investigations into closed-loop algorithms for individualized interventions may greatly improve the reliability of those interventions. This is particularly important in a clinical setting where the aim is to optimize symptom improvement.

To conclude, we have demonstrated a more efficient research process, taking as a working model the field of brain stimulation to overcome the problem of selecting stimulation parameters for each individual. The method we suggest here can be extended with minimal or no changes to different fields in which the optimal parameters are unknown and/or expensive to assess, including drug discovery, invasive and non-invasive brain stimulation, and physical and mental training in both typical and atypical populations.

## Methods

### Subjects and ethics statement

Fifty subjects gave written consent before the start of the study. All met the safety criteria for transcranial electrical stimulation (tES) and received financial compensation of £20. In addition to this compensation, subjects had the chance of winning an additional £50 based on their performance (the winner was randomly drawn from the best 10 performers over the three blocks). Behavioral data from all 49 subjects aged between 18–30 years old (31 of whom were female) were used for the pBO (mean age = 22.52 ± standard deviation (SD) = 4.09). All were right-handed. One completed their education at GCSE level, 14 at A-level, 17 were undergraduates and 18 were postgraduates. In the UK educational system GCSE refers to secondary education and A-level refers to an advanced level that can lead to university. All subjects reported no contraindications to electrical stimulation or any history of dyscalculia, dyslexia or attentional deficits. The proposed study received ethical approval from The University of Oxford Medical Sciences Interdivisional Research Ethics Committee (protocol number: MSD-IDREC-C2-2014-033). Additionally, we pre-registered the present study on the Open Science Framework; see https://osf.io/bg2pd.

### Overview of experimental paradigm and stimuli

The study was conducted in an electrically shielded lab space at the University of Oxford, where environmental influences (i.e., lighting, seating, EEG cap) were kept constant throughout the experiment. Over the course of the experiment, subjects completed four blocks of fifty multiplication problems—one baseline block and three stimulation blocks (see **Fig 2**). After recording an initial 4-minute baseline resting state EEG, the task was explained to the subjects and they completed 10 practice trials of approximately 4 minutes, followed by the baseline block of 10 minutes during which no tACS was administered. In each block, subjects had to indicate which answer was correct as accurately and as fast as possible with no time limit present (see **Fig 2A**). Subjects indicated the correct answer by pressing either the left or the right button on a response box situated in front of them. They underwent three blocks of multiplications of 30 minutes in total in which they received tACS. Prior to each tACS block, the pBO algorithm was run (<5 seconds) to determine the stimulation parameters (current intensity and frequency) to be delivered during the upcoming experimental block based on the individual subject’s performance in the baseline block. The stimulation parameters were automatically selected by the algorithm and administered whilst maintaining blinding in both the subject and experimenter (for a complete list of the applied current and frequency, see **S1** and **S2 Tables**). Rs-EEG of 4 minutes was recorded again after each stimulation block. Note that the stimulation electrodes were integrated in the EEG cap which allowed us to use the same electrodes for both EEG recording and stimulation.

### Behavioral stimuli

Arithmetic performance was tested using an arithmetic calculation paradigm, consisting of problems involving a single-digit number multiplied by a two-digit number, with a three-digit outcome. This paradigm was presented using Matlab’s psychtoolbox version 3. A calculation paradigm was used instead of a retrieval paradigm since calculation has been associated with an increased activation in the frontoparietal network [30,56,57]. None of the multiplications included operands with the digits 0, 1, or 2 to prevent variations in difficulty. In addition, the two-digit operand was not smaller than 15, did not use repeated digits, and was not a multiple of 10. Subjects were visually presented with a multiplication problem on a screen with a correct and incorrect answer positioned under the multiplication problem on the left and right side. The position of the correct and incorrect answer was randomly allocated to the right and left sides of the screen and they always differed by 10. Each problem was presented only once, and their order was randomized.

### Measurement of baseline abilities

An arithmetic baseline task containing 50 different arithmetic multiplications was presented to measure individual arithmetic ability in terms of response times and accuracy. Subsequently, baseline drift rates were calculated for each subject according to the two-choice EZ-diffusion model [40]. This approach allowed us to dissect the different components in the chain of information processing by modeling the decision process and targeting the cognitive component of interest (the drift rate, which reflects ability and task difficulty by modeling response time and accuracy), rather than auxiliary components [40,58]. This model was chosen to combine reliably the response time and accuracy in one outcome that could be optimized through the pBO procedure. The 50 trials completed in the baseline block were randomly divided into two sets, and for both sets a separate drift rate was calculated. One was used as a measure of the subject’s baseline ability, whilst the other was used to normalize the drift rates calculated during the optimization phase (e.g., during the experimental procedure of the pBO). This was done to eliminate dependency between the subject’s baseline ability score and the normalized score in each stimulation block [59]. To reduce fatigue, subjects had a break of 30 s after every 10 trials. After completing the baseline task, subjects had a short break (~3 minutes) before they continued with the experimental procedure of the pBO.

### Experimental procedure of the pBO

Before the start of the pBO procedure, a burn-in phase was used that consisted of 60 random tACS parameters assigned to the first 20 subjects to initiate the pBO procedure. The pBO code (i.e., coded stimulation parameters, for example, 1 mA and 10 Hz has been coded as 320) was manually initiated before each performance block and took <5 seconds to run. The stimulation parameters selected by the algorithm, based on an individual’s baseline ability, were automatically saved and passed to the stimulation software to maintain double blinding. The pBO algorithm selected stimulation parameters for the subject, with the aim of improving behavioral performance given their baseline ability. This was done in an iterative process across 30 subjects, with the algorithm’s estimate of the optimal stimulation parameter, at any given baseline ability, becoming more accurate as more subjects were tested. At each run of the pBO algorithm, all previously collected data was used, including data collected in the burn-in phase and the GP was refitted to model all the data. As we *a priori* defined in our preregistration, we utilized a pre-set stopping criteria of 50 subjects, after which testing was ceased.

Our rationale to set the sample size to 50 subjects was as follows: For BO without noise [60], n = 10–20 per dimension is often used. For BO with noise, a recent work set the number of evaluations to 25 per dimension [61]. However, to take into account the possibility that we might deal with increased noise in the present study, we set it to n = 50 per dimension (50 subjects x 3 blocks each, equals 150 evaluations to account for three dimensions (frequency, current, and baseline ability).

In total, 150 diverse multiplication problems (three blocks of 50 trials) were administered during the experimental procedure. After each block, performance drift rates were calculated immediately, another rs-EEG was measured for four minutes, and then for the next block the combination of tACS parameters (frequency and current) was changed. Thus, behavioral performance optimization relied on the frequency and current of tACS together with the baseline cognitive ability as indicated by the drift rate. Each subject received three different frequency-current tACS combinations.

### Transcranial alternating current stimulation

The alternating current stimulation was administered over the left frontoparietal network (see **Fig 2C**). The tACS was delivered via two stimulation (3.14 mm diameter) NG Pistim Ag/AgCl electrodes (F3 and P3) with one return electrode (Cz) using the Starstim 32 (Neuroelectrics, Barcelona). The conductive interface used was electrode gel Signagel. The impedances of the electrodes were held at < 10 kΩ. The stimulation intensity ranged between 0.1–1.6 mA peak-to-peak in steps of 0.1 for the burn-in phase of the study. For the optimization phase, 0 mA was added to control for possible sham influences. We chose the maximum stimulation intensity based on a small pilot study on three subjects to determine the maximum comfortable intensity. Different combinations of frequencies in the range from 5–50 Hz and intensity in the range from 0–2 mA peak-to-peak were tested. Subjects indicated by means of verbal communication their highest tolerable intensity, where 1.6 mA was chosen as the maximum intensity. The stimulation frequency ranged between 5–50 Hz in steps of 1 Hz for the whole experiment.

Stimulation was administered in a double-blind manner during the three experimental blocks with a maximum of 10 minutes for each block. Stimulation started 45 s before the start of the block and changed after every block. If the subjects received a stimulation intensity of 0 mA during a block (sham stimulation), a ramp-up and a ramp-down of 30 s was initiated to provide the initial skin sensations during stimulation to ensure blinding. When the subject completed a block within 10 minutes, stimulation was ramped down for 30 s and the subject proceeded to the four-minute rs-EEG. Note that in cases where subjects completed the task in under 10 minutes, they did not receive the full length of stimulation. These stimulation blocks did not differ from the stimulation block in which the subject received the full length of stimulation except for performance. Twenty-four of the 150 stimulation blocks had a duration of less than 10 minutes but more than 7.50 minutes, and 126 stimulation blocks had a duration of more than 10 minutes. This posed no problem, since the present study only investigated the online effects of tACS on arithmetic behavior. After completing a block related to one tACS combination, the subjects filled out a questionnaire in which they were asked several questions designed to gauge the level of sensation experienced during stimulation (see S1 Questionnaire Items). We used this data to assess the relationship between the intensity rating of every sensation and tACS amplitude.

### Resting state-EEG recordings and pre-processing

Resting state-EEG recordings were made at the start of the study (before baseline measurements) and immediately after every stimulation block. Electrophysiological data were obtained with eight gel Ag/AgCl electrodes (F3, P3, F4, P4, Fz, Cz, Pz, AF8) according to the international 10/10 EEG system using the wireless Starstim R32 sensor system (Neuroelectrics, Barcelona, Spain), with no online filters. The conductive interface used was electrode gel Signagel. The ground consisted of adhesive active common mode sense (CMS) and passive driven right leg (DRL) electrodes which were positioned on the right mastoid. All EEG measurements had a duration of four minutes in which the subjects had their eyes open while watching a fixation point in the middle of the screen. Raw EEG data were recorded and used for offline analysis using EEGLAB 13.6.5b [62], which is an open source toolbox running on Matlab R2018b [63]. Data were high-pass filtered at 0.1 Hz, and low-pass filtered at 50 Hz (using a finite impulse response filter). Visual inspection was carried out to remove artefacts caused by muscle movement. We rejected an EEG recording from analysis if more than 25 percent of the data in a given block were removed. This resulted in the EEG data from four stimulation blocks being rejected (2% of the data). Independent component analysis was used to remove blinks and noisy components. On average, 1.23 ± 0.62 SD components were rejected per subject, with a maximum of three components and a minimum of zero.

Note that our EEG recording and preprocessing pipeline was unsuited to analyzing gamma frequency activity for a number of reasons. Firstly, we used a conservative pipeline to ensure the removal of noise from our data instead of removing relevant brain activity. Additionally, the short recording time of 4 minutes used in this study is inadequate to reliably measure gamma activity [64], as well as the unavoidable use of resting recordings leading to a lack of task-evoked gamma peak, which would be much more reliably detected than resting state [65]. However, we would like to reinstate that the EEG outcomes are not the main focus of the present study.

### Sensation analysis

In line with a previous study [66], and as stated in our pre-registration, we expected higher sensitivity ratings of the tACS parameters with higher current values compared with lower currents. Therefore, we predicted a positive correlation between the intensity rating for itching, pain, burning, phosphenes, warmth, and fatigue. We performed a separate correlation analysis to calculate the bivariate Pearson’s coefficient (r) or Spearman’s rho (r_s_) depending on normality to assess the relationship between the intensity of different sensations induced by tACS and height of tACS current (**S2** and **S4 Figs**).

### Bayesian optimization

Bayesian optimization uses *f* to denote an unknown objective function (e.g., black-box function) for which we do not have a closed-form expression, but we could have an infinite number of queries. Furthermore, this black-box function is expensive and time costly to evaluate. Formally, let *f*: *X*→*R* (*R* is the set of all real numbers, representing the values from −∞ to + ∞) be a well-behaved function, defined on a subset *X* ⊆ *R*^*d*^ whereby d is the number of dimensions. The standard BO approach is aimed at solving the following global optimization problem:

$$x^{*}=arg\underset{x\in X}{max}\;f(x)$$ (1)

The BO algorithm is aimed at finding the global optimum of arithmetic performance, as indicated by drift rates, of the black-box function *f*(*x*) by making a series of evaluations at *x*_1_, *x*_2_,…,*x*_*T*_ (see S1 Mathematical Variables).

### Personalized Bayesian optimization

While our approach could personalize a given treatment based on any individual characteristic, such as neural or biometric data, we chose cognitive ability, as the literature provides more supporting evidence for its moderating effect [1–3,7,8,46,67], especially as it is closely related to our desired behavioral outcome. Each subject has their individual arithmetic baseline ability: this value is considered as the personalized value. We needed to measure this value *p* separately for every subject, as was done during the baseline task. We expected to see that the optimal parameters will vary with different baseline abilities. That is, the optimal parameter x* depends on the different values of *p*. For this reason, the standard BO presented in the previous section may not have been appropriate. Therefore, we proposed to solve the following optimization problem, defined formally as:

$$x^{*}(p)=arg\underset{x\in X}{max}\;f(x,p)$$ (2)

where *p* is the baselines ability given for each subject. The optimal parameter x* is not defined globally, but specifically to a variable *p*. This is the key difference of our pBO in comparison with the standard BO, while we acknowledge related research in BO with environmental variables [21,68,69].

### Objective function

In the present tACS study, we used the following objective function *f*(*x*,*p*):

$$f(x,p)=\frac{Vstim}{Vbase}$$

where *Vstim* is the drift rate during the 50 trials of an arithmetic multiplication block normalized by the *Vbase* (the drift rate calculated over 25 random trials from the baseline task). To determine improvement between the baseline and the stimulation block, a second *Vbase* was calculated over the other 25 trials from the baseline task. A Pearson correlation was calculated to determine if the two drift rates from the baseline and the one used for the improvement index are related (*r* = 57, p < .001). The acquisition functions are carefully designed to allow a trade-off between exploration of the search space and the exploitation of current promising regions. A burn-in phase of 60 random tACS frequency-current combinations was used. These were assigned to the first 20 subjects of the BO design to determine the amount of variation induced by stimulation. We decided to use a large burn-in in our paradigm to design a reliable BO algorithm that was based on a large amount of data.

### Personalized Gaussian process for joint modeling of target function and baseline ability

Standard BO models *f* with a GP, *f*~*GP*(*m*, *k*), where *m* is the mean function and *k* is the covariance function [69]. This flexible distribution allowed us to associate a normally distributed random variable at every point in the continuous input space. Therefore, we obtained the predictive distribution for ***f*** at a new observation *x* that also follows a Gaussian distribution. Its mean (*μ*) and variance (*σ*^2^) are given by:

$$\mu (x\prime )=k(x^{\prime };X)K{\left(X;X\right)}^{-1}y$$

$$\sigma^{2}(x\prime )=k(x\prime ;x\prime )-k(x^{\prime };X)K{\left(X;X\right)}^{-1}k{\left(x^{\prime };X\right)}^{T}$$ (3)

where *K*(*U*; *V*) is a covariance matrix whose element (*i*; *j*) is calculated as *k*_*i*,*j*_ = *k*(*x*_*i*_; *x*_*j*_) with *x*_*i*_ ∈ *U* and *x*_*j*_ ∈ *V*. Behavioral observations are typically associated with noise that can be accommodated in a GP model. Namely, every *f*(*x*) processes extra variance due to independent noise:

$$y_{i}=f(x_{i})+\epsilon_{i}\;\mathrm{where}\;\epsilon_{i}\sim N(0,\sigma_{n}^{2})\;\mathrm{and}\;\sigma_{n}^{2}\;\mathrm{is}\;\mathrm{the}\;\mathrm{noise}\;\mathrm{variance}.$$ (4)

When considering noise, the output follows the GP as $y∼GP(m,k+\sigma_{n}^{2}\delta_{i,j})$, where *δ*_*i*,*j*_ = 1 if *i* = *j* is the Kronecker’s delta. The covariance function for a noisy process becomes the sum of the signal covariance and the noise covariance. Specifically, the exponentiated-quadratic covariance function between two observations can be computed as:

$$k(x_{i};\;x_{j})=exp(-\frac{{\left(x_{i}-x_{j}\right)}^{2}}{2\sigma_{l}^{2}})+\sigma_{n}^{2}\delta_{i,j}$$ (5)

For a more elaborate overview of GPs, we refer the interested reader to Rasmussen and Williams [70].

In our personalized setting, one of the possible solutions is to build a GP and optimization for each value *p*. However, such a simplistic approach faces a critical problem of data efficiency, because the number of data samples is not sufficient to estimate each value *p* separately. Therefore, we extended the GP surrogate to jointly model our target function *f* and the additional personalized dimension *p*, rather than using a separate GP for every subject. Specifically, the GP covariance becomes:

$$k\left(\{x_{i},p_{i}\};\{x_{j},p_{j}\}\right)=k(x_{i},x_{j})\times k(p_{i},p_{j})$$ (6)

where *k*(*x*_*i*_, *x*_*j*_) is defined in Eq (5) and $k(p_{i},p_{j})=exp(-\frac{{\left(p_{i}-p_{j}\right)}^{2}}{2\sigma_{p}^{2}})$. These covariance functions correspond to the parameters and baselines, respectively. We note that the length scale parameter $\sigma_{p}^{2}$ used in *k*(*p*_*i*_, *p*_*j*_) is different from $\sigma_{x}^{2}$ used in *k*(*x*_*i*_, *x*_*j*_). For example, if the baseline ability length-scale $\sigma_{p}^{2}$ is extremely large, it means the performance is not changing with respect to the baseline performance. On the other hand, if $\sigma_{p}^{2}$ is small, it means the performance function is changing rapidly with the baseline performance. We later maximized the marginal likelihood to estimate these length scale parameters directly from the data [70]. Under our modification for the GP, we could estimate the predictive mean and predictive variance:

$$\mu (x,p)=k(\{x,p\};Z)K{\left(Z;Z\right)}^{-1}y$$

$$\sigma^{2}(x,p)=k(\{x,p\};\{x,p\})-k(\{x,p\};Z)K{\left(Z;Z\right)}^{-1}k{\left(\{x,p\};Z\right)}^{T}$$ (7)

Where we denoted *Z* = [*X*, *P*], the personalized covariance matrix *k* is defined in Eq (6).

### Acquisition function

To select the next point to evaluate, the acquisition function *α*(*x*) was chosen to construct a utility function based on the GP surrogate model mentioned above. Instead of maximizing the expensive original function *f*, we maximized the cheaper acquisition function to select the next most optimal point:

$$x_{t+1}=arg\underset{x\in X}{max}\;\alpha (x)$$

In this auxiliary maximization problem, the acquisition function form is known and can be easily optimized by standard numerical techniques. One of the most common choices for the acquisition function is the GP upper confidence bound (GP-UCB):

α(x,p)=μ(x,p)+κ×σ(x,p)

where *μ*(*x*, *p*) and *σ*(*x*, *p*) are the GP predictive mean and variance defined in Eq (7) and *κ* is the hyperparameter controlling the exploration-exploitation trade-off. One can follow Srinivas et al. to specify the value of *κ* to achieve the theoretically-guaranteed performance [69]. The second common acquisition function is the expected improvement (EI) [43]. The EI finds the next sampling point given the highest chance of expectation to improve upon the best-found value so far. Using the analytical expression of Gaussian distribution, we have the EI in closed-form as:

$$\alpha^{EI}(x,p)=[\mu (x,p)-f^{+}]\times \Phi (z)+\sigma (x,p)\times \phi (z)$$

where $z=\frac{\mu (x,p)-f^{+}}{\sigma (x,p)}$ and *f*^+^ is the best observed value up to the current iteration, *Φ*(*z*) is the standard normal cumulative distribution function and *ϕ*(*z*) is the standard normal probability density function. When the uncertainty is zero *σ*(*x*, *p*) = 0, the *α*^*EI*^(*x*,*p*) = 0.

### Hyperparameters considerations

Personalized Bayesian optimization relies on a personalized Gaussian process surrogate model to select a next point for testing. This personalized GP model (defined in section ‘Personalized Gaussian process for joint modeling of target function and baseline ability’.) involves several hyperparameters including the length scales *σ*_*l*_ in Eq (5), *σ*_*p*_ in Eq (6), and the noise variance $\sigma_{n}^{2}$ in Eq (4). We make use of the property of the Gaussian process to estimate these hyperparameters directly from the observed data by maximizing the log marginal likelihood of a GP model [70]. For robustness, we have also normalized the input *x* ∈ [0,1]^2^, *p* ∈ [0,1] and standardized the output score *N*(0,1) as popularly used in previous work [71]. Given this normalized space, the estimated hyperparameters vary by iterations within the range as follows *σ*_*l*_ ∈ [0.03,0.4], *σ*_*p*_ ∈ [0.07,0.5] and $\sigma_{n}^{2}\in [0.01,2]$.

One can optimize the EI [43,72] over the current best result or the GP-UCB. In short, it is more likely that the UCB selects evaluations with both a high mean and high variance. The EI and UCB have been shown to be efficient in the number of function evaluations required to find the global optimum of many multimodal black-box functions [43,73]. During the present study, the EI was applied to find the optimum in arithmetic performance. Lastly, we decided to remove one extreme drift rate value of 3.6 during the experimental procedure due to possible ceiling effects of the BO for sampling the same stimulation parameters. However, the inclusion of this data point did not significantly alter our results. For similar results without exclusion of this data point, see **S5 Fig**. In total, we acquired 148/150 iterations. In addition, due to technical problems another data point was not included in the BO procedure.

### Simulation analysis

Simulations were run to validate the pBO procedure during arithmetic performance and tACS. This analysis aimed to show that pBO can outperform both a ‘standard’ BO algorithm and random sampling when identifying an optima in a noisy environment. Note that the present study contained three dimensions, namely frequency, current, and individualized baseline ability. Therefore, we utilized a Hartmann function [42] that included four local minima in three dimensions as an example, to enable our simulations to be comparable to our experimental data. As human-based studies are prone to noisy evaluations, we decided to introduce noise in the simulation by running the same Hartmann 3-dimensional function whilst adding different noise variation values ($\sigma_{n}^{2}$). The pBO algorithm presented in this work was compared to a standard BO algorithm which did not incorporate the personalized dimension into its evaluations, as well as a random sampling algorithm. Performance in these simulations was compared in terms of the best found value at each of the 60 iterations, as well as the distance from the known optima location with the Euclidean distance as a metric. Each simulation was repeated 30 times at each level of noise, and the three algorithm’s mean performance and standard deviation over these repeats was calculated. Note that it is not possible to calculate the Euclidean distance between subsequent stimulation pairs due to the inclusion of a personalized variable.

### EEG analysis of spectral power and frontoparietal theta connectivity

The rs-EEG data of the remaining datasets were separated in 2 second segments with an overlap of 1 second and windowed with a Hann window. Subsequently, data were transformed into the frequency domain via fast Fourier transformation (FFT). Theta (4–8 Hz) and beta (14–30 Hz) frequency bands were calculated according to their relative power (μV^2^) and normalized by dividing the absolute frequency power of each frequency band by the average absolute power in the 1.5–30 Hz range. In addition, we also normalized the power by dividing the absolute frequency power by the average absolute power in the 4–50 Hz range. The weighted phase lag index (wPLI) in the theta and beta range was computed to determine the phase lag synchronization between the left frontal and parietal areas at baseline and after every tACS block. This computation was made for the complementary channels F3 and P3. The theta wPLI was calculated for 4–8 Hz in steps of 1 Hz and the beta wPLI was calculated from 14–30 Hz in steps of 4 Hz. Furthermore, we normalized wPLI by calculating the wPLI at the applied tACS frequency divided by the baseline wPLI at the same frequency.

First, outliers were removed with Cook’s distance before running statistical models. To focus on the relation between arithmetic baseline ability and spectral power, separate regression models were run with theta and beta power as dependent factors. Likewise, we tested whether there was a relation between frontoparietal theta and beta connectivity scores by running several regression models in steps of 1 Hz for theta wPLI and steps of 4 Hz for beta wPLI.

### Statistical analysis

All the reported inferential statistical analysis was done with RStudio version 1.2.5042 with significance defined as *p* ≤ 0.05 [74]. All data is presented as mean ± SD with n = 50 for electrophysiology analysis and n = 49 for the pBO analysis. Pre-processing of electrophysiological data was done with EEGLAB 13.6.5b [62] which is an open source toolbox running on MatlabR2018b [63]. Subsequently, normalized electrophysiological data was checked for outliers with Cook’s distance and entered in log-transformed regression models using the stats package [74] with spectral power or connectivity measures as dependent variables and arithmetic baseline ability as independent variable. A correlation analysis on normally distributed datasets was run to calculate the bivariate Pearson’s coefficient (r) to investigate differences in sensation and blinding, and a non-parametric (Spearman’s rho (r_s_)) correlation on non-normally distributed variables. Generalized linear mixed effects models (GLMM) were run with the nlme package [75] to explore EEG changes induced by tACS during arithmetic performance (see **S1 Text**). The pBO algorithm and simulations were run with Python version 3.6 [76], using the SciPy, NumPy and Scikit-learn libraries [77–79]. Note that no inferential statistics such as a GLMM is able to reliably investigate performance gains due to the inability to disentangle the exploration and exploitation trade-off of the pBO algorithm between blocks.

## Supporting information

S1 Fig Histogram plot showing the number of subjects in every baseline ability (n = 50). More subjects were on the lower part of the spectrum of the baseline ability range than the higher part.
(TIF)

S2 Fig Side effects of different tACS current intensities. Different side-effects are shown according to the indicated sensation on a scale from 1–10 (n = 150 based on 50 subjects). 1 was indicated as a low sensation (‘I did not feel the sensation’) and 10 is a strong sensation (‘I felt the sensation to a considerable degree’). The high value for fatigue at 0.1 mA is likely to be due to the low number of subjects (n = 2) who received this stimulation, and it might reflect a general state.
(TIF)

S3 Fig Side effects of different tACS frequency intensities. Different side-effects are shown according to the indicated sensation on a scale from 1–10 (n = 150 based on 50 subjects). 1 was indicated as a low sensation (‘I did not feel the sensation’) and 10 is a strong sensation (‘I felt the sensation to a considerable degree’).
(TIF)

S4 Fig Blinding efficacy of tACS. The figure shows the percentage of correct indications that stimulation was real for every applied current (n = 150 based on 50 subjects). Blinding efficacy of tACS is at change level (~50%).
(TIF)

S5 Fig Best found value of the personalized Bayesian optimization (pBO) procedure without exclusion of data point 46 (n = 50). Arithmetic performance in terms of drift rate for every best-found value for *f*(x) and for every iteration of the pBO procedure during stimulation without exclusion of data point number 46.
(TIF)

S6 Fig The interaction between EEG power, current, and frequency in predicting arithmetic performance for subjects with low arithmetic baseline ability (n = 25). Arithmetic performance (log transformed drift rates) during stimulation is shown on the y-axis and the normalized (post stimulation/pre stimulation) EEG power μV^2^/Hz (log transformed) based on the applied tACS frequency after stimulation is shown on the x-axis for four different tACS frequencies (4 Hz, 15 Hz, 30 Hz, and 50 Hz). Current intensity is indicated by the blue line (0.1 mA), the black line (1 mA), and the grey line (1.6 mA). Shaded areas indicate 95% confidence intervals. Note that different tACS categories and current intensities are presented for visualization purposes, to allow a better grasp of an interaction that is based on continuous variables.
(TIF)

S1 Table Number of subjects (n) according to the different currents (mA) received in one stimulation block. (DOCX)

S2 Table Number of subjects (n) according to the different frequencies (Hz) received in one stimulation block. (DOCX)

S3 Table Fixed effects of the mixed effects model for arithmetic performance in low ability subjects (n = 25). Note: **p < 0.05; **p <0.01.
(DOCX)

S4 Table Fixed effects of the mixed effects model for arithmetic performance for high ability subjects (n = 24). Note: **p < 0.05; **p < 0.01.
(DOCX)

S1 Text Supplementary results and discussion. (DOCX)

S1 Mathematical Variables All mathematical variables used in the present study clarified. (DOCX)

S1 Questionnaire Questionnaire items used to assess sensation levels during stimulation. (DOCX)
