# Supplementary figures and images for: Personalized brain stimulation for effective neurointervention across participants
Source: PLoS Comput Biol. 2021 Sep 9;17(9):e1008886. doi: 10.1371/journal.pcbi.1008886 (PMC8454957; doi:10.1371/journal.pcbi.1008886)

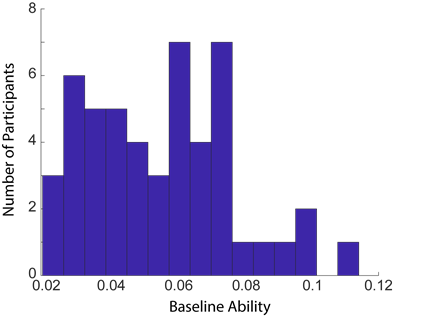

Supplement: S1 Fig — More subjects were on the lower part of the spectrum of the baseline ability range than the higher part. (TIF) [file pcbi.1008886.s001.tif]

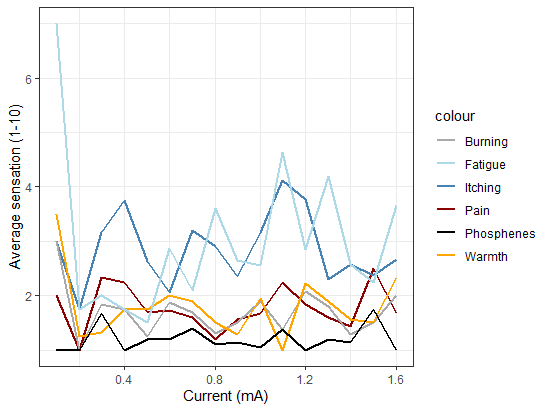

Supplement: S2 Fig — Different side-effects are shown according to the indicated sensation on a scale from 1–10 (n = 150 based on 50 subjects). 1 was indicated as a low sensation (‘I did not feel the sensation’) and 10 is a strong sensation (‘I felt the sensation to a considerable degree’). The high value for fatigue at 0.1 mA is likely to be due to the low number of subjects (n = 2) who received this stimulation, and it might reflect a general state. (TIF) [file pcbi.1008886.s002.tif]

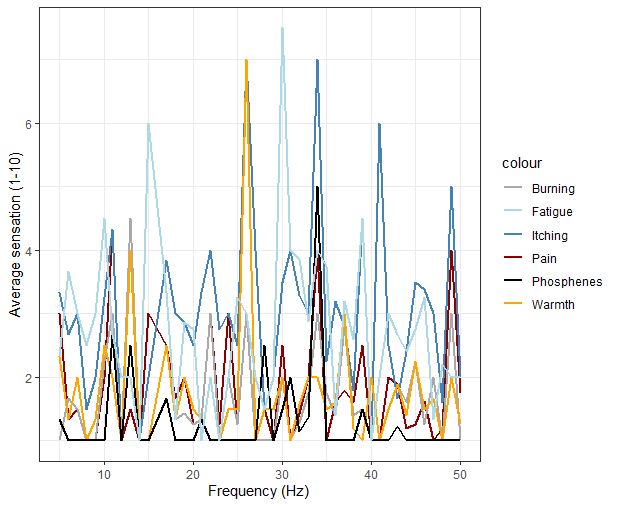

Supplement: S3 Fig — Different side-effects are shown according to the indicated sensation on a scale from 1–10 (n = 150 based on 50 subjects). 1 was indicated as a low sensation (‘I did not feel the sensation’) and 10 is a strong sensation (‘I felt the sensation to a considerable degree’). (TIF) [file pcbi.1008886.s003.tif]

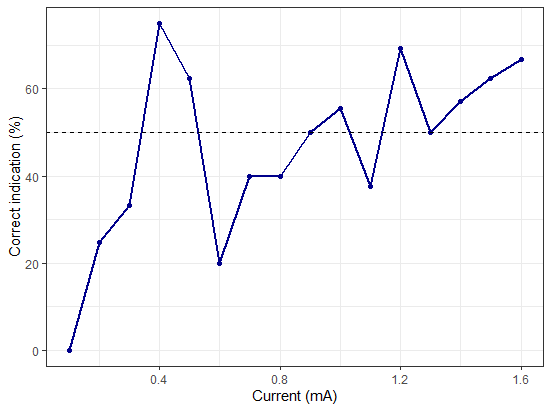

Supplement: S4 Fig — The figure shows the percentage of correct indications that stimulation was real for every applied current (n = 150 based on 50 subjects). Blinding efficacy of tACS is at change level (~50%). (TIF) [file pcbi.1008886.s004.tif]

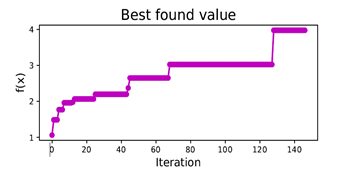

Supplement: S5 Fig — Arithmetic performance in terms of drift rate for every best-found value for f(x) and for every iteration of the pBO procedure during stimulation without exclusion of data point number 46. (TIF) [file pcbi.1008886.s005.tif]

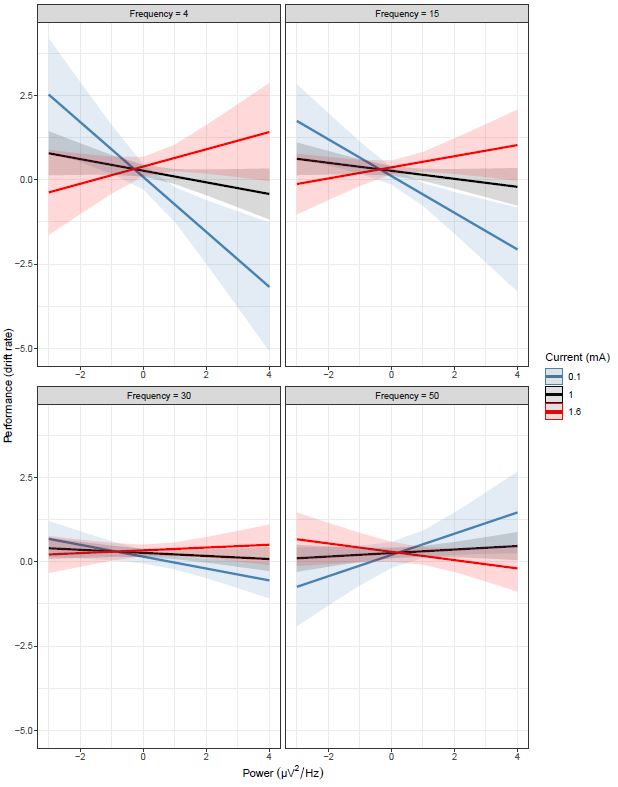

Supplement: S6 Fig — Arithmetic performance (log transformed drift rates) during stimulation is shown on the y-axis and the normalized (post stimulation/pre stimulation) EEG power μV2/Hz (log transformed) based on the applied tACS frequency after stimulation is shown on the x-axis for four different tACS frequencies (4 Hz, 15 Hz, 30 Hz, and 50 Hz). Current intensity is indicated by the blue line (0.1 mA), the black line (1 mA), and the grey line (1.6 mA). Shaded areas indicate 95% confidence intervals. Note that different tACS categories and current intensities are presented for visualization purposes, to allow a better grasp of an interaction that is based on continuous variables. (TIF) [file pcbi.1008886.s006.tif]
