# Supplementary material for: Personalized brain stimulation for effective neurointervention across participants
Source: PLoS Comput Biol. 2021 Sep 9;17(9):e1008886. doi: 10.1371/journal.pcbi.1008886 (PMC8454957; doi:10.1371/journal.pcbi.1008886)
Supplement: S1 Table — (DOCX) [file pcbi.1008886.s007.docx]

| **Current (mA)** | **N (number of subjects)** |
| --- | --- |
| 0 | 0 |
| 0.1 | 2 |
| 0.2 | 4 |
| 0.3 | 6 |
| 0.4 | 4 |
| 0.5 | 16 |
| 0.6 | 15 |
| 0.7 | 10 |
| 0.8 | 10 |
| 0.9 | 14 |
| 1.0 | 18 |
| 1.1 | 8 |
| 1.2 | 13 |
| 1.3 | 10 |
| 1.4 | 7 |
| 1.5 | 8 |
| 1.6 | 3 |
