# Supplementary material for: Personalized brain stimulation for effective neurointervention across participants
Source: PLoS Comput Biol. 2021 Sep 9;17(9):e1008886. doi: 10.1371/journal.pcbi.1008886 (PMC8454957; doi:10.1371/journal.pcbi.1008886)
Supplement: S2 Table — (DOCX) [file pcbi.1008886.s008.docx]

| **Frequencies (Hz)** | **N (number of subjects)** | **Frequencies (Hz)** | **N (number of subjects)** |
| --- | --- | --- | --- |
| 5 | 3 | 29 | 2 |
| 6 | 3 | 30 | 2 |
| 7 | 2 | 31 | 1 |
| 8 | 2 | 32 | 7 |
| 9 | 3 | 33 | 8 |
| 10 | 4 | 34 | 1 |
| 11 | 3 | 35 | 4 |
| 12 | 1 | 36 | 5 |
| 13 | 2 | 37 | 5 |
| 14 | 1 | 38 | 4 |
| 15 | 1 | 39 | 1 |
| 17 | 6 | 40 | 1 |
| 18 | 3 | 41 | 1 |
| 19 | 7 | 42 | 2 |
| 20 | 4 | 43 | 9 |
| 21 | 3 | 44 | 5 |
| 22 | 1 | 45 | 4 |
| 23 | 4 | 46 | 8 |
| 24 | 2 | 47 | 3 |
| 25 | 4 | 48 | 4 |
| 26 | 1 | 49 | 1 |
| 27 | 1 | 50 | 3 |
| 28 | 2 |  |  |
