# Supplementary material for: Personalized brain stimulation for effective neurointervention across participants
Source: PLoS Comput Biol. 2021 Sep 9;17(9):e1008886. doi: 10.1371/journal.pcbi.1008886 (PMC8454957; doi:10.1371/journal.pcbi.1008886)
Supplement: S3 Table — Note: **p < 0.05; **p <0.01. (DOCX) [file pcbi.1008886.s009.docx]

| **Predictors** | **Estimates** | **CI (95%)** | **df** | **t-value** | **p-value** |
| --- | --- | --- | --- | --- | --- |
| (Intercept) | 0.04 | -0.43 – 0.52 | 36 | 0.20 | 0.83 |
| EEG power | -0.99 | -1.63 – -0.35 | 36 | -3.13 | 0.003** |
| tACS current | 0.21 | -0.22 – 0.66 | 36 | 1.00 | 0.31 |
| tACS frequency | 0.002 | -0.01 – 0.02 | 36 | 0.36 | 0.71 |
| Power x current | 0.80 | 0.19 – 1.41 | 36 | 2.66 | 0.01* |
| Power x frequency | 0.02 | 0.01 – 0.05 | 36 | 2.83 | 0.007** |
| Current x frequency | -0.003 | -0.02 – 0.01 | 36 | -0.40 | 0.68 |
| Power x current x frequency | -0.02 | -0.04 – -0.00 | 36 | -2.53 | 0.01* |
