# Supplementary material for: Personalized brain stimulation for effective neurointervention across participants
Source: PLoS Comput Biol. 2021 Sep 9;17(9):e1008886. doi: 10.1371/journal.pcbi.1008886 (PMC8454957; doi:10.1371/journal.pcbi.1008886)
Supplement: S4 Table — Note: **p < 0.05; **p < 0.01. (DOCX) [file pcbi.1008886.s010.docx]

| **Predictors** | **Estimates** | **CI (95%)** | **df** | **t-value** | **p-value** |
| --- | --- | --- | --- | --- | --- |
| (Intercept) | 0.05 | -0.37 – 0.47 | 38 | 0.24 | 0.80 |
| Power | 0.53 | -0.08 – 1.16 | 38 | 1.75 | 0.08 |
| tACS current | 0.17 | -0.34 – 0.70 | 38 | 0.68 | 0.49 |
| tACS frequency | 0.004 | -0.01 – 0.02 | 38 | 0.73 | 0.46 |
| Power x current | -0.51 | -1.27 – 0.25 | 38 | -1.36 | 0.17 |
| Power x frequency | -0.01 | -0.03 – 0.00 | 38 | -1.78 | 0.08 |
| Current x frequency | -0.006 | -0.02 – 0.01 | 38 | -0.88 | 0.38 |
| Power x current x frequency | 0.01 | -0.01 – 0.04 | 38 | 1.32 | 0.19 |
