# Supplementary material for: Personalized brain stimulation for effective neurointervention across participants
Source: PLoS Comput Biol. 2021 Sep 9;17(9):e1008886. doi: 10.1371/journal.pcbi.1008886 (PMC8454957; doi:10.1371/journal.pcbi.1008886)
Supplement: S1 Text — (DOCX) [file pcbi.1008886.s011.docx]

**Supplementary Results and Discussion**

**|** Histogram plot showing the number of subjects in every baseline ability (n = 50).

More subjects were on the lower part of the spectrum of the baseline ability range than the higher part.

**Sensations and Blinding of tACS**

All subjects reported a low level of sensation during stimulation, with no serious adverse side effects, which complies with the previous literature [1]. Interestingly, itching and fatigue were more frequently reported than sensations such as burning, pain, phosphenes, and warmth (**S2** and **S3 Figs**). No reliable correlations were found between the intensity ratings for all sensations and tACS amplitude (all *p* >.10). Moreover, the blinding efficacy of tACS was at chance level (**S4 Fig**). However, the correct indication of real stimulation increased with the current of the applied stimulation.

**EEG and Arithmetic Performance**

As stated in our pre-registration, we also investigated EEG changes induced by tACS during arithmetic performance (see **S1** and **S2 Tables**). To do this reliably, we computed power (electrode Fz) and wPLI scores (electrodes F3 and P3) for each tACS block and normalized these values according to the baseline rs-EEG values. This normalization was undertaken to exclude possible frequency band specific power changes induced by stimulation at a fixed frequency. For example, stimulation at 10 Hz could lead to an increased entrainment in the 10 Hz frequency band [2]. A four-way interaction was found between arithmetic baseline ability, EEG power, current, and frequency when predicting arithmetic performance (SE = 0.01, df = 71, t = 3.48, *p* < .001). To look more closely at this interaction, we investigated the interaction between EEG power, current, and frequency in subjects with low and high baseline abilities separately in a mixed effects model by performing a median split (median = -2.85). We revealed a 3-way interaction for subjects with low baseline ability (n = 25) (**S3 Table** and **S6 Fig**). In contrast, for high baseline ability subjects (n = 24), there was no three-way interaction present (all *p* > .08) (**S4 Table**). In short, for low ability subjects, a high current (1.6 mA) leads to a steeper increase in arithmetic performance and EEG power when frequencies are low (4 Hz) (**S6 Fig**). This pattern decreased as frequency increased and flipped to a decreased performance and power when the applied tACS frequency was in the 50 Hz frequency range. When running the same analysis for connectivity, no three-way interaction was found for low and high ability subjects (*p* > .05).

Our electrophysiological findings after stimulation indicated an interaction between tACS parameters, oscillatory brain activity, and arithmetic performance for subjects with low baseline ability (**S3 Table**). Interestingly, this interaction between brain stimulation and brain activity was not found for high baseline ability subjects. Our preferred explanation for this finding is that tACS strongly entrains neural oscillations when there is a high-performance gain due to low baseline ability. However, when looking at our group-level pBO model (**Fig 3**) there are no differential effects of tACS on performance levels between subjects with low and high baseline ability. Neural entrainment due to tACS could possibly serve as a compensatory mechanism to improve performance for subjects with low baseline ability [3].

**References**

1. Matsumoto H, Ugawa Y. Adverse events of tDCS and tACS: A review. Clin Neurophysiol Pract [Internet]. 2017 Jan 1 [cited 2019 Aug 28];2:19–25. Available from: https://www.sciencedirect.com/science/article/pii/S2467981X16300233

2. Helfrich RF, Schneider TR, Rach S, Trautmann-Lengsfeld SA, Engel AK, Herrmann CS. Entrainment of brain oscillations by transcranial alternating current stimulation. Curr Biol. 2014 Feb 3;24(3):333–9.

3. Witkowski M, Garcia-Cossio E, Chander BS, Braun C, Birbaumer N, Robinson SE, et al. Mapping entrained brain oscillations during transcranial alternating current stimulation (tACS). Neuroimage. 2016 Oct 15;140:89–98.
