# Supplementary material for: Personalized brain stimulation for effective neurointervention across participants
Source: PLoS Comput Biol. 2021 Sep 9;17(9):e1008886. doi: 10.1371/journal.pcbi.1008886 (PMC8454957; doi:10.1371/journal.pcbi.1008886)
Supplement: S1 Mathematical Variables — (DOCX) [file pcbi.1008886.s012.docx]

**Mathematical variables**

$\boldsymbol{\sigma}_{\boldsymbol{n}}^{\boldsymbol{2}}\boldsymbol{\in[0.01,2]}$: measurement noise variance

$\boldsymbol{f}\left( \boldsymbol{x} \right)\boldsymbol{:}$ this is the regular black-box function which takes an input $x\in R^{d}$ and returns an output $y\in R$.

$\boldsymbol{f(x,p)}$: this is a personalized black-box function which takes the personalized score $p$ and the input variable $\boldsymbol{x}$. The personalized score is different from the input variable that we can modify the input $\boldsymbol{x}$ to optimize the function while we cannot change the personalized score $p$.

$\boldsymbol{GP}\left( \boldsymbol{m,k} \right)\boldsymbol{:}$ a Gaussian process model which takes a mean function $m$ and a covariance function $k$.

A covariance between two input data point $x_{i},x_{j}$ is defined as follows:

$$\boldsymbol{k}\left( \boldsymbol{x}_{\boldsymbol{i}}\boldsymbol{,}\boldsymbol{x}_{\boldsymbol{j}} \right)\boldsymbol{=exp}\left( \boldsymbol{-}\frac{\left( \boldsymbol{x}_{\boldsymbol{i}}\boldsymbol{-}\boldsymbol{x}_{\boldsymbol{j}} \right)^{\boldsymbol{2}}}{\boldsymbol{2}\boldsymbol{\sigma}_{\boldsymbol{l}}^{\boldsymbol{2}}} \right)\boldsymbol{+}\boldsymbol{\sigma}_{\boldsymbol{n}}^{\boldsymbol{2}}\boldsymbol{\delta}_{\boldsymbol{i,j}}$$

$\boldsymbol{\sigma}_{\boldsymbol{l}}\boldsymbol{\in[0.03,0.4]}$**:** the lengthscale parameter of the input variables.

A covariance between two personalized data point $p_{i},p_{j}$ is defined as follows:

$$\boldsymbol{k}\left( \boldsymbol{x}_{\boldsymbol{i}}\boldsymbol{,}\boldsymbol{x}_{\boldsymbol{j}} \right)\boldsymbol{=exp}\left( \boldsymbol{-}\frac{\left( \boldsymbol{p}_{\boldsymbol{i}}\boldsymbol{-}\boldsymbol{p}_{\boldsymbol{j}} \right)^{\boldsymbol{2}}}{\boldsymbol{2}\boldsymbol{\sigma}_{\boldsymbol{p}}^{\boldsymbol{2}}} \right)$$

$\boldsymbol{\sigma}_{\boldsymbol{p}}\boldsymbol{\in[0.07,0.5]}$**:** the lengthscale parameter of the personalized scores.

$\boldsymbol{\mu}\left( \boldsymbol{x,p} \right)\boldsymbol{:}$the predictive mean of a Gaussian process at the input $\boldsymbol{x}$ given the personalized score $p$

$\boldsymbol{\sigma}^{\boldsymbol{2}}\left( \boldsymbol{x,p} \right)\boldsymbol{:}$the predictive variance of a Gaussian process at the input $\boldsymbol{x}$ given the personalized score $p$

$\kappa$: hyperparameter controlling the exploration-exploitation in GP-UCB acquisition function.

$f^{+}:$ the best observed value so far used in Expected improvement (EI) acquisition function.
