# Supplementary material for: Personalized brain stimulation for effective neurointervention across participants
Source: PLoS Comput Biol. 2021 Sep 9;17(9):e1008886. doi: 10.1371/journal.pcbi.1008886 (PMC8454957; doi:10.1371/journal.pcbi.1008886)
Supplement: S1 Questionnaire — (DOCX) [file pcbi.1008886.s013.docx]

**Questionnaire Items:** Sensation levels experienced during stimulation

After every block in which the subject received stimulation, the following items were presented:

Do you believe that you received real or placebo stimulation?

1. Real
2. Placebo
3. I do not know

Please indicate whether you experienced any discomfort during the stimulation by typing the corresponding number:
            1 = None (I did not feel the sensation)
            2-3 = Mild (I mildly felt the sensation)
            4-6 = Moderate (I felt the sensation)
            7-10 = Strong (I felt the sensation to a considerable degree)
 for Pain, Burning, Warmth/Heat, Fatigue/Decreased alertness, Flashing lights

In the case of perceived sensations: How much did these sensations affect your general state?
           1 = Not at all
            2 = Slightly
           3 = Considerably
            4 = Much
           5 = Very much

How long did the sensations last? (0 to 10 minutes)?
